# Delayed neutralizing antibody response in the acute phase correlates with severe progression of COVID-19

**DOI:** 10.1038/s41598-021-96143-8

**Published:** 2021-08-16

**Authors:** Hitoshi Kawasuji, Yoshitomo Morinaga, Hideki Tani, Miyuki Kimura, Hiroshi Yamada, Yoshihiro Yoshida, Yusuke Takegoshi, Makito Kaneda, Yushi Murai, Kou Kimoto, Akitoshi Ueno, Yuki Miyajima, Koyomi Kawago, Yasutaka Fukui, Ippei Sakamaki, Yoshihiro Yamamoto

**Affiliations:** 1 Department of Clinical Infectious Diseases, Graduate School of Medicine and Pharmaceutical Sciences, University of Toyama, 2630 Sugitani, Toyama, 930-0194 Japan; 2 Department of Microbiology, Graduate School of Medicine and Pharmaceutical Sciences, University of Toyama, 2630 Sugitani, Toyama, 930-0194 Japan; 3 Department of Virology, Toyama Institute of Health, 17-1 Nakataikoyama, Imizu-shi, Toyama, 939-0363 Japan

**Keywords:** SARS-CoV-2, Antibodies, Viral infection

## Abstract

Adaptive immunity to severe acute respiratory syndrome coronavirus 2 (SARS-CoV-2) dynamics remain largely unknown. The neutralizing antibody (NAb) levels in patients with coronavirus disease 2019 (COVID-19) are helpful for understanding the pathology. Using SARS-CoV-2 pseudotyped virus, serum sample neutralization values in symptomatic COVID-19 patients were measured using the chemiluminescence reduction neutralization test (CRNT). At least two sequential serum samples collected during hospitalization were analyzed to assess NAbs neutralizing activity dynamics at different time points. Of the 11 patients, four (36.4%), six (54.5%), and one (9.1%) had moderate, severe, and critical disease, respectively. Fifty percent neutralization (N50%-CRNT) was observed upon admission in 90.9% (10/11); all patients acquired neutralizing activity 2–12 days after onset. In patients with moderate disease, neutralization was observed at earliest within two days after symptom onset. In patients with severe-to-critical disease, neutralization activity increased, plateauing 9–16 days after onset. Neutralization activity on admission was significantly higher in patients with moderate disease than in patients with severe-to-critical disease (relative % of infectivity, 6.4% vs. 41.1%; *P* = .011). Neutralization activity on admission inversely correlated with disease severity. The rapid NAb response may play a crucial role in preventing the progression of COVID-19.

## Introduction

Understanding the dynamics and key features of adaptive immunity to severe acute respiratory syndrome coronavirus 2 (SARS-CoV-2) is essential for controlling the coronavirus disease 2019 (COVID-19) pandemic; however, the details of the emergence, establishment, and long-lasting memory of adaptive immunity in response to SARS-CoV-2 remain largely unknown. Antibodies are important elements of the adaptive immune response. Recent evidence shows that recovered COVID-19 patients can generate antibodies, albeit at variable levels, that specifically bind to various structural proteins of SARS-CoV-2 particles shortly after the onset of disease^1–3^. Among these virus-specific antibodies, only those capable of blocking SARS-CoV-2 spike (S) protein-mediated viral attachment and/or entry of host cells, called neutralizing antibodies (NAbs), play an important role in virus clearance and have been considered as key immune products in the protection against or treatment of viral diseases. Severe COVID-19 can be associated with high neutralization titers in the sub-acute or later phases^4,5^; however, whether the acquisition of neutralizing antibodies in the acute phase is related to disease severity is unclear.

The level of NAbs has also been used as a gold standard for evaluating the efficacy of vaccines. However, the measurement of NAb levels is needed to deal with infectious virus and active cells, which makes it a difficult procedure with respect to the problems of standardization and the need for specialized laboratory equipment with BSL-3 facilities.

To circumvent the need for BSL-3 laboratories, we recently developed a fundamental NAbs detection method for SARS-CoV-2 based on the chemiluminescence reduction neutralization test (CRNT) and VSV-based pseudotyped viruses with SARS-CoV-2 S (Sfullpv) or truncated S proteins (St19pv) to evaluate the expression and neutralization values of NAbs against SARS-CoV-2^6^.

In this study, at least two sequential serum samples collected on admission and during hospitalization from confirmed symptomatic COVID-19 patients with different disease severities were analyzed to assess the dynamics of the neutralizing activities of NAbs at different time points and determine the clinical significance of the measurement results using our fundamental NAbs detection method.

## Material and methods

### Study design

We performed a single-center study at the Toyama University Hospital. Hospitalized adult symptomatic COVID-19 patients with different disease severities, in which at least two sequential serum samples were collected during hospitalization, were included in this study. The patients were tested for SARS-CoV-2 RNA in the serum and nasopharyngeal (NP) swabs collected on or around admission. Given that viral culture was not performed to determine the viability of the virus, the presence of viral RNA in the serum is referred to as RNAemia instead of viremia^7^. To study the dynamics of the neutralizing antibody response, blood samples from symptomatic patients were collected on admission, and subsequently when clinically needed during hospitalization.

### Data collection

A retrospective chart review was performed for all individuals in the study to identify basic demographic data, medical history, clinical presentation, and hospital admission. Severity was divided into four categories: mild (symptomatic patients without pneumonia), moderate (pneumonia patients without required oxygen supplementation), severe (requiring oxygen supplementation), and critical (requiring invasive mechanical ventilation, presence of shock and/or multiple organ dysfunction).

### Neutralization assay

Neutralization of the human serum against pseudotyped viruses was measured based on a previous report^6^. Briefly, Vero cells were treated with two 100-fold dilutions of sera from in-hospital patients with COVID-19 and then inoculated with pseudotyped VSVs bearing the S protein (Sfullpv), the 19-amino acid-truncated S protein of SARS-CoV-2 (St19pv) or VSV-G (VSVpv). The infectivity of the pseudotyped viruses was determined by measuring luciferase activity after 24 h of incubation at 37 °C, based on our preliminary study^6^, and the number of days after admission or onset until the first result of 50% neutralization in the CRNT (N50%-CRNT) was recorded.

### Statistical analysis

Continuous and categorical variables are presented as median [interquartile range (IQR)] and n (%), respectively. We used the Mann–Whitney U test, χ^2^ test, or Fisher’s exact test to compare the differences between the patients with moderate and severe/critical disease. Data were analyzed using JMP Pro version 15.0.0 software (SAS Institute Inc., Cary, NC, USA). The time courses of the neutralization activities were also analyzed using nonlinear regression in JMP Pro version 15.0.0 software (SAS Institute Inc., Cary, NC, USA).

### Ethical approval

This study was performed in accordance with the Helsinki Declaration and was approved by the ethical review board of the University of Toyama (approval No.: R2019167). Written informed consent was obtained from all patients***.***

## Results

Of the 11 in-hospital symptomatic patients enrolled in this study, ten patients recovered and were discharged from the hospital. The median age of patients was 78 years (range, 50–100 years); three (27.3%) patients were male, and eight (72.7%) patients were female. Five (45.5%), two (18.2%), and one (9.1%) patients had a history of hypertension, cardiovascular disease, and diabetes, respectively. Four patients (36.4%) had moderate disease, six (54.5%) had severe disease, and the remaining patients (9.1%) had critical disease. First serum samples of all patients were collected at the time of hospital presentation and all patients were subsequently admitted on the same day of hospital presentation. For the neutralization test of the first sample, N50%-CRNT was observed on admission in 90.9% (10/11) of all patients and thereafter observed in all patients from 6 and 12 days after admission and onset, respectively. The median time from onset to first achievement of N50%-CRNT was 8 days (range, 2–12 days).

**Figure 1:**
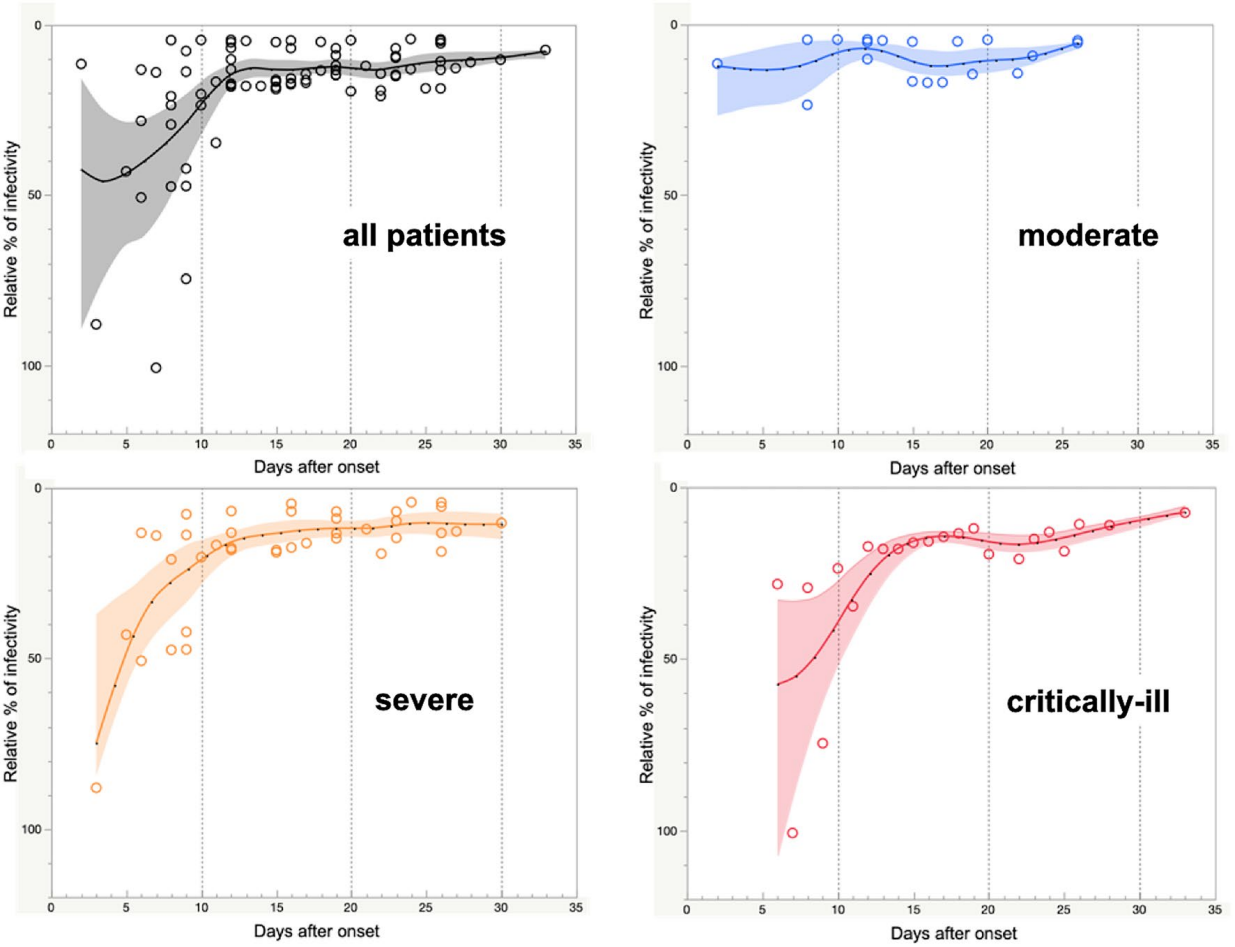
Trends in neutralization activities in symptomatic patients stratified by severity. Nonlinear regression models of all eleven patients (black), four moderate (blue), six severe (orange), and one critically ill patients (red). The models were calculated using the mean data of three independent assays of the patients stratified by severity. Solid curves are spline curves fitted to the mean with a λ of 0.05. Shading indicates the 95% confidence interval of each model.

Based on the sequential analysis during hospitalization, a total of 79 serum samples from 11 patients were assessed by measuring the neutralization activities. The median number of CRNTs performed in each patient was seven (range, 2–22), and the number of days between the CRNT and onset ranged from 2 to 33 days (Fig. 1 and Fig. S1). The median length of hospital stay was 46 days (range, 14–58 days) (Table 1). Regarding the evaluation of the kinetics of neutralization activities, there were different characteristics according to disease severity. In patients with moderate disease (Fig. 1), the degree of neutralization activity in the first samples collected on admission was high, and neutralization was observed two days after symptom onset in patient #11 (Supplemental Fig. 1). In contrast, in severely critically ill patients, the degree of neutralization activity in the first samples collected on admission was relatively low and gradually increased to reach a plateau approximately 9–16 days after onset. Notably, when comparing neutralization activities on admission between patients with moderate and severe-to-critical disease, the degree of neutralization activity of St19pv in the moderate patients was significantly higher than that in the patients with severe-to-critical disease (6.4 ± 3.4% vs. 41.1 ± 24.6%; *P* = 0.011) (Table 1). No significant differences were seen between patients with moderate and severe-to-critical disease in the time from onset to hospital presentation (9 days vs. 6 days; *P* = 0.39) (Table 1). With respect to age, there were no significant differences in neutralizing antibody activity on admission, time from onset to first achievement of N50%-CRNT, and disease severity between patients ≥ 80 and < 80 years old, respectively (*P* = 0.65, 1 and 0.55).

**Table 1:** Demographic, clinical characteristics, results of SARS-CoV-2 test, and neutralization antibody activities on admission of eleven patients stratified by severity.

Characteristics	Overall patients, n = 11	Moderate, n = 4	Severe-to-critical, n = 7	*P*-value
Age, median, years	78	59.5	83	.13
**Sex**				
Male, n (%)	3 (27.3)	1 (25.0)	2 (28.6)	1
**Comorbidities, n (%)**				
Hypertension	5 (45.5)	1 (25.0)	4 (57.1)	.55
Cardiovascular disease	2 (18.2)	0 (0.0)	2 (28.6)	.49
Diabetes	1 (9.0)	0 (0.0)	1 (14.3)	1
**SARS-CoV-2 test**				
Viral load (log_10_ copies/μL) of nasopharyngeal swab collected around admission, median (IQR)	2.6 (2.1–4.9)	1.5 (0.7–4.5)	3.1 (2.1–4.9)	.40
Presence of RNAemia on admission, n (%)	5 (45.5)	1 (25.0)	4 (57.1)	.55
**Neutralization test of the first sample obtained on admission**				
Number of patients in whom neutralization activity reached ≥ 50% even on admission, n (%)	10 (90.9)	4 (100.0)	6 (85.7)	1
Median days after onset (range)	8 (2–12)	9 (2–12)	6 (3–9)	.39
Relative % of infectivity, mean ± SD	28.5 ± 26.0	6.4 ± 3.4	41.1 ± 24.6	.011
Median time between neutralization tests (range)	7 (2–22)	3.5 (3–8)	8 (2–22)	.21
Length of hospital stay, median days (IQR)	46 (19–51)	20 (15.3–39.8)	50 (22–56)	.073
**Treatment, n (%)**				
Favipiravir	9 (81.8)	4 (100.0)	5 (71.4)	.49
Antibiotic therapy	7 (63.6)	0 (0.0)	7 (100.0)	.0003
**Clinical outcomes, n (%)**				
Required oxygen supplementation	7 (63.6)	0 (0.0)	7 (100.0)	.003
ICU admission	6 (54.5)	0 (0.0)	6 (85.7)	.015
Invasive mechanical ventilation	1 (9.1)	0 (0.0)	1 (14.3)	1
In-hospital mortality	1 (9.1)	0 (0.0)	1 (14.3)	1

## Discussion

It is still unknown when sufficient immunity against SARS-CoV-2 is obtained during the course of an infection. In the present study, we confirmed that patients who recovered from COVID-19 acquired neutralizing activity 2–12 days after onset based on CRNT. However, it is not known which type of antibody predominantly plays a role in neutralization because the CRNT result reflects the comprehensive action of a variety of antibodies. In a recent study on COVID-19, Sterlin et al.^8^ suggested that neutralizing capacity rapidly increased and plateaued by day 10 post-symptom onset, which is consistent with our results for patients with moderate and severe disease. They also indicated the possibility that early SARS-CoV-2-specific humoral responses were dominated by IgA. Another study reported that seroconversion occurred 2 days after symptom onset^9^, supporting the early acquisition of neutralizing activity in patient #11 (Fig. S1).

To date, the relationship between disease severity and the magnitude of the SARS-CoV-2-specific NAbs responses in patients with COVID-19 is largely unknown. A recent study demonstrated a positive correlation between the magnitude of NAbs responses and disease severity; in particular, the highest NAbs capacity in the sera was found in patients with severe disease who recovered from COVID-19^1^. These results were not consistent with our results, but this discrepancy may be attributed to the different phases, the acute and convalescent phases, in which the NAbs response was measured.

In the acute phase on admission, our study found that the degree of neutralization activity in patients with moderate disease was significantly higher than that in patients with severe-to-critical disease. It has been reported that NAbs appear earlier at higher levels in patients with severe or moderate disease than in those with mild disease^10^; however, our findings clearly indicate the difference in the induction of the neutralizing response between moderate and severe-to-critical disease. These results suggest that the acquisition of NAbs in the early stages of infection may be related to disease severity. Because severity positively correlates with SARS-CoV-2 RNAemia^7^, the acquisition of NAbs, including mucosal immunity, can modify early COVID-19 pathology. A recent study demonstrated that the development of plasma antibodies was correlated with decreases in viral RNAemia, consistent with potential humoral immune clearance of virus^11^. Although the low number of severe-critical cases were involved and the variability of the measures was observed in the present study, the degree of neutralization activity in the acute phase may reflect the ability to clearance of virus from circulation or other sites in the body.

In contrast, in the convalescent phase in COVID-19 patients, high NAbs capacity in elderly patients and/or those with severe disease as previously reported^1,12^, which is mainly due to specific IgG, could be a result of the severe inflammatory response. Therefore, the specific background related to the ability of NAbs should be verified.

Our results indicate a potential role for the measurement of NAb levels at the time of hospital presentation in the early detection of individuals at risk of severe clinical illness and in guide for effective use of therapeutic convalescent plasma products and recombinant antibodies targeting SARS-CoV-2. Additionaly, it may also be useful to assess NAbs neutralizing activity dynamics after vaccination at different time points for determining the most suitable vaccination schedule.

This study had several limitations. First, the number of involved patients was very small and selected using consecutive instead of random sampling. Thus, we had insufficient power to incorporate confounding factors, representativeness is relatively insufficient, and the samples could only represent the general situation to a certain extent. Second, the subjects ranged in age from 50 to 100 years, mainly had moderate and severe disease, and only one critically ill patient was included. Although there was no difference in neutralizing activity with age, indicating potential transferability to younger age groups, the NAb response in younger or asymptomatic infections and mild patients requires further exploration. Third, in the severe subjects and the critically-ill subject, variability of the measures was observed at the early timepoints. In our evaluation of the performance of the CRNT assay, the values of samples with strong neutralization are more stable than those samples with moderate to weak ones. In addition, because the severe and critically-ill patients can be dramatically changed in their circulatory dynamics, the serum concentration of antibody can be affected. Thus, two factors can be involved in the values during the acute phase, and in that case, it may be necessary to evaluate neutralization activities under various dilution conditions. Fourth, the meaning of the quantitative values of CRNT results remains controversial. To make the value clinically meaningful, continuous improvement of our method and comparison with clinical findings are required.

## Conclusions

In conclusion, this study showed that neutralizing antibody activities were observed in all patients with symptomatic COVID-19, even in the early stage of illness, and the degree of neutralization activity on admission was significantly higher in patients with moderate disease than in those with severe-to-critical disease. The present study highlights that a rapid NAb response may play a crucial role in preventing severe progression of COVID-19.

## Supplementary Information

Supplementary Figure S1.
