# Supplementary material for: Delayed neutralizing antibody response in the acute phase correlates with severe progression of COVID-19
Source: Sci Rep. 2021 Aug 16;11:16535. doi: 10.1038/s41598-021-96143-8 (PMC8368204; doi:10.1038/s41598-021-96143-8)

***Supplementary Information***

***Title***

Delayed neutralizing antibody response in the acute phase correlates with severe progression of COVID-19

***Authors***

Hitoshi Kawasuji^1^, Yoshitomo Morinaga^2^, Hideki Tani^3^, Miyuki Kimura^2^, Hiroshi Yamada^2^, Yoshihiro Yoshida^2^, Yusuke Takegoshi^1^, Makito Kaneda^1^, Yushi Murai^1^, Kou Kimoto^1^, Akitoshi Ueno^1^, Yuki Miyajima^1^, Koyomi Kawago^1^, Yasutaka Fukui^1^, Ippei Sakamaki^1^, Yoshihiro Yamamoto^1#^

***Affiliation***

1. Department of Clinical Infectious Diseases, Toyama University Graduate School of Medicine and Pharmaceutical Sciences, University of Toyama

2. Department of Microbiology, Toyama University Graduate School of Medicine and Pharmaceutical Sciences, University of Toyama

3. Department of Virology, Toyama Institute of Health

**# Correspondence:** Yoshihiro Yamamoto, MD, PhD

Department of Clinical Infectious Diseases, Graduate School of Medicine and Pharmaceutical Sciences, University of Toyama

2630 Sugitani, Toyama, 930-0194, Japan.

Tel: 076-434-7245, E-mail: [yamamoto@med.u-toyama.ac.jp](mailto:yamamoto@med.u-toyama.ac.jp)

**Figure S1.**

Neutralization of St19pv and VSVpv infections through sera from eleven hospitalized patients #1–#11 with COVID-19.

The pseudotyped viruses were preincubated with two a hundred-fold dilution of sera from eleven (#1–#11) hospitalized COVID-19 patients, including four moderate (#2, #3, #10 and #11), six severe (#1, #4, #6, #7, #8 and #9), and one critically ill patients (#5). Thereafter, Vero cells were infected with pseudotyped VSVs bearing the 19-amino acid-truncated S protein of SARS-CoV-2 (red) or VSV-G (blue). Infectivities of pseudotyped viruses were determined by measuring luciferase activities 24 h post-infection. The results are from three independent assays with error bars representing standard deviations.


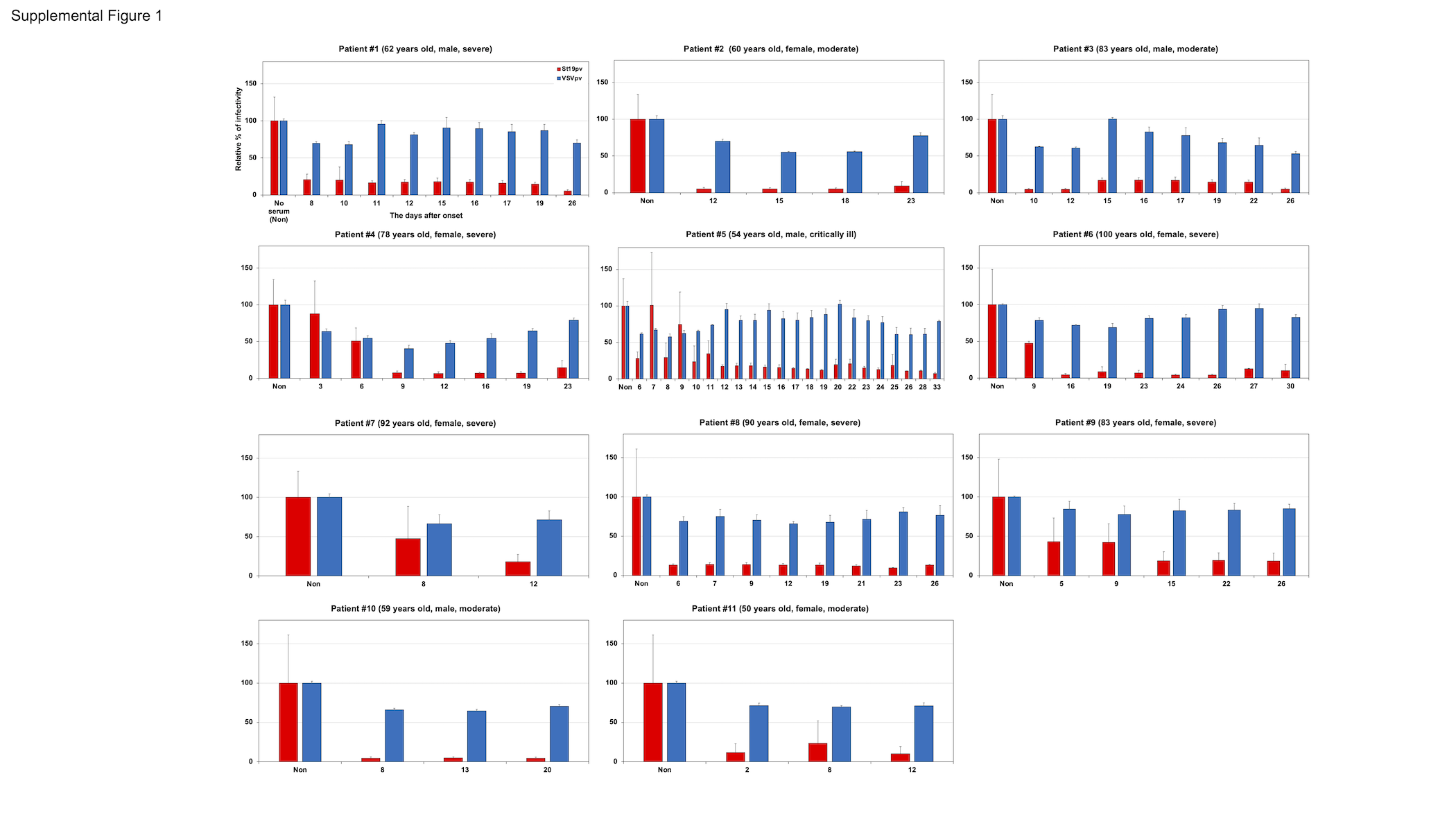

Supplement: Supplementary file 1 — Supplementary Figure S1. [file 41598_2021_96143_MOESM1_ESM.docx]
